# Non-truncating *BMPR1A* variants associated with familial colorectal cancer and adenomatous polyps

**DOI:** 10.1186/s12885-025-14865-8

**Published:** 2025-09-29

**Authors:** Taina T. Nieminen, Outi Kuismin, Riitta Laine, Anna Lepistö, Laura Koskenvuo, Laura Renkonen-Sinisalo, Markus J. Mäkinen, Ari Ristimäki, Jukka-Pekka Mecklin, Päivi Peltomäki

**Affiliations:** 1 Department of Medical and Clinical Genetics, Medicum, University of Helsinki, Helsinki, Finland; 2 Department of Clinical Genetics, Research Unit of Clinical Medicine, Medical Research Center Oulu, Oulu University Hospital, University of Oulu, Oulu, Finland; 3 Department of Abdominal Surgery, Helsinki University Hospital, Helsinki, Finland; 4 Applied Tumor Genomics, Research Programs Unit, University of Helsinki, Helsinki, Finland; 5 Translational Medicine Research Unit, Medical Research Center Oulu, Oulu University Hospital, University of Oulu, Oulu, Finland; 6 Department of Pathology, HUS Diagnostic Center, HUSLAB, Helsinki University Hospital, University of Helsinki, Helsinki, Finland; 7 Department of Education & Research, The Wellbeing Services of Central Finland, Jyväskylä, Finland; 8 Department of Sports & Health Sciences, Jyväskylä University, Jyväskylä, Finland; 9 HUSLAB Laboratory of Genetics, HUS Diagnostic Center, HUS, Helsinki University Hospital, Helsinki, Finland

**Keywords:** Colorectal cancer, Adenomatous polyp, Familial colorectal cancer type x, Juvenile polyposis syndrome, *BMPR1A*

## Abstract

**Background:**

Pathogenic variants of the bone morphogenetic protein receptor type 1 A (*BMPR1A*) gene underlie juvenile polyposis syndrome (JPS), a rare autosomal dominant condition characterized by multiple gastrointestinal hamartomatous polyps. Recent findings indicate that constitutional *BMPR1A* variants can also be associated with various non-JPS phenotypes without hamartomatous polyps. The basis of varying genotype - phenotype relationships is poorly understood.

**Methods:**

We investigated four families with non-truncating variants of *BMPR1A* affecting different functional domains. Clinical presentation resembled familial colorectal cancer type X-like syndrome with dominantly inherited microsatellite-stable gastrointestinal adenomas and carcinomas. To gain insights into genotype-phenotype associations, exome sequencing was conducted on normal and tumor tissue DNAs. Constitutional *BMPR1A* variants underwent a thorough evaluation for clinical significance, by, e.g., co-segregation analyses and in silico modeling, supplemented by haplotyping and genealogical studies. All available tumors were examined for histology and molecularly for *BMPR1A* “second hits” and mutational signatures.

**Results:**

Targeted sequencing of blood DNA revealed a three-nucleotide deletion (*BMPR1A* c.264_266 del) in one family, a three-nucleotide insertion (*BMPR1A* c.506_507insTCC) in two families, and a missense change (*BMPR1A* c.766G > A) in a fourth family. The two families with *BMPR1A* c.506_507insTCC had a shared ancestral origin. Co-segregation of the variants with colorectal cancer and/or polyps, in-silico modeling, and two hit inactivation by loss of heterozygosity or somatic point mutations in tumors, together with the absence of other possible predisposing variants by exome sequencing, supported the idea of tumor predisposition being attributable to the *BMPR1A* variants. Polyps examined from variant carriers had adenomatous histology, except for three polyps with hamartomatous features, originating from two *BMPR1A* carriers from two families. While no hamartoma samples were available for molecular investigation, somatic mutational profiles of colorectal adenomas and carcinomas resembled those of mismatch repair-proficient colorectal tumors in general.

**Conclusions:**

Our findings support the notion that the clinical phenotype of *BMPR1A* variants may extend beyond classical JPS. Genotype-phenotype correlations are complex, since molecular comparison of constitutional and tumor features of our families to those published from JPS families in the literature show a significant overlap. The variety of clinical phenotypes warrants recognition in the clinical management of *BMPR1A* carriers and their family members.

**Supplementary Information:**

The online version contains supplementary material available at 10.1186/s12885-025-14865-8.

## Introduction

Bone morphogenetic protein (BMP) signaling is one of four main pathways that control normal epithelial cell differentiation [1]. The ligands, BMPs, are members of the TGF β superfamily and bind to their receptors, BMPR1A and BMPR1B (type I) or BMPR2 (type II), that are transmembrane serine/threonine kinases. BMP binding to type II receptor results in phosphorylation of type I receptors, which subsequently phosphorylates SMAD1, SMAD5, and SMAD8. Their association with SMAD4 results in nuclear localization and activation of BMP/TGF β signaling.

BMP signaling acts as a tumor suppressor and promotes apoptosis in mature colonic epithelial cells [2]. Constitutional loss-of-function variants of the *BMPR1A* and *SMAD4* genes underlie predisposition to the juvenile polyposis syndrome (JPS), a dominantly inherited susceptibility to multiple juvenile polyps in the gastrointestinal tract and increased risk of colorectal cancer and to a lesser extent, certain other cancers [3]. Moreover, the BMP pathway is inactivated by acquired genetic or epigenetic alterations in up to 80% of sporadic colon cancers [4]– [5]. Polyps developing in JPS patients are hamartomatous polyps with expanded mesenchymal stroma [2]. Contrary to adenomatous polyps, epithelial cells of juvenile polyps show no dysplasia. *BMPR1A*- and *SMAD4*-associated tumorigenesis is postulated to follow the “landscaper” model where an abnormal stromal environment leads to neoplastic transformation of the adjacent epithelium [6].

We previously described two large families segregating constitutional *BMPR1A* variants (c.264_266del p.(Glu88del) in one family and c.68 − 10_68 + 14del p.(Gly23GlufsX10) in the other family) but with adenomatous rather than hamartomatous polyps [7]. Clinically, these families complied with familial non-polyposis colorectal cancer with DNA mismatch repair (MMR) proficient tumors. We now provide updated clinical and molecular data on the previous family with *BMPR1A* c.264_266del and describe three new families with non-truncating constitutional *BMPR1A* variants and clinical and histological presentation other than JPS.

## Materials and methods

### Patients and samples

This study was based on four Finnish non-polypotic colorectal cancer families with MMR-proficient tumors. HNPCC20 met the Amsterdam criteria [8]– [9] and complied with familial colorectal cancer type X (FCCTX), FCCX-U and FCCX-V jointly fulfilled the Amsterdam criteria without age requirement, and FCCX-W met the Bethesda criteria [10]. All families revealed heterozygous non-truncating variants in *BMPR1A* by targeted sequencing performed in the research setting (HNPCC20 [7]) or as part of a diagnostic workout (FCCX-U, FCCX-V, and FCCX-W). Following the identification of the *BMPR1A* variant in the index individual(s), diagnostic testing by accredited panels encompassing known colorectal cancer susceptibility genes (*APC*, *BMPR1A*, *MLH1*, *MLH3*, *MSH2*, *MSH6*, *MUTYH*, *PMS2*, *POLD1*, *POLE*, *SMAD4* ja *STK11*, and additional genes depending on the panel) was offered to the remaining family members. The families had no pathogenic constitutional variants in *MLH1*, *MSH2*, *MSH6*, and *PMS2* and no evidence of microsatellite instability (MSI) or aberrant MMR protein expression in tumor tissues. Histological diagnoses, originally provided in routine diagnostic service, were re-evaluated by gastrointestinal pathologists (MJM and AR). Areas with high percentages of tumor cells were selected and used for DNA extraction by the method of Isola et al. (1994) [11].

### Exome sequencing

Institute for Molecular Medicine Finland, FIMM (Helsinki, Finland) carried out exome sequencing with 100–200x mean target coverage. DNA samples from HNPCC20 were sequenced with Illumina NovaSeq S4 PE151 system using Twist_Core_Exome_RefSeq_targets_hg19.gff library (33 Mb). A variant calling pipeline (VCP 3.7b) developed by FIMM [12] was used for primary and secondary data analysis. Paired-end reads were aligned to the GRCh37/Hg19 human genome build by using the Burrows-Wheeler Alignment.

DNA samples from FCCX-V, FCCX-U, and FCCX-W were sequenced with Illumina NovaSeq 6000 system (Illumina, San Diego, CA, USA). For library preparation and exome capture, 50 ng of blood gDNA and 50–200 ng of formalin-fixed paraffin-embedded (FFPE) gDNA was processed according to Twist Library Preparation EF 2.0 with Enzymatic Fragmentation DOC-001239 REV 1.0 and Twist Target Enrichment Protocol DOC-001085 REV 2.0 (Twist Bioscience, San Francisco, CA, USA) with following modifications. Library preparation was optimized for some of the FFPE samples: 200 ng input, 20 min fragmentation, 9 pre-PCR cycles. Unique Dual Index UMI oligos by IDT (Integrated DNA Technologies, Coralville, IA, USA) were used as ligation adapters. Library quantification and quality check was performed using LabChip GX Touch HT High Sensitivity assay (PerkinElmer, USA) and Qubit Broad Range DNA Assay (Thermo Fisher Scientific, Waltham, MA, USA). Libraries were pooled to 7-plex, 8-plex or 9-plex reactions according to concentration (Labchip). The exome enrichment was performed using Twist Comprehensive exome probes (37 Mb). The captured library pools were quantified for sequencing using Collibri Library Quantification kit (Thermo Fisher Scientific, Waltham, MA, USA) and Agilent 2100 Bioanalyzer High Sensitivity DNA kit (Agilent, Santa Clara, CA, USA). Sequencing was performed with Illumina NovaSeq 6000 system using S4 flow cell with lane divider and v1.5 chemistry. Read length for the paired-end run was trimmed to 101 + 8 + 8 + 101. Illumina DRAGEN Bio-IT Platform v3.9 [13] was used for primary and secondary data analysis against GRCh38 as the human reference.

### Exome data analysis to detect constitutional variants

VarSeq^®^ (Golden Helix Inc., Bozeman, MT) was used for tertiary analysis. We applied several filters to exclude common variants and select significant variants. Sample based computed filter for variants was first set to be PASS. Variants with total minor allele frequencies (MAF) *≥* 0.001 according to GnomAD v4.0 were removed. RefSeq Genes 110, NCBI database was used to extract indel, misssense and nonsense variants from only protein coding area, excluding intronic and intergenic variants but including splice site-related intronic variants. Then dbNSPF Functional Prediction Voting was used to predict missense variants’ pathogenicity. There are five different prediction programs in VarSeq: SIFT Pred, Mutation Taster Pred, MutationAssessor Pred, FATHMM Pred, and FATHMM MKL Coding Pred. We only considered missense type variants predicted pathogenic by 5 of 5 prediction programs. Variants in low complexity regions were excluded by using Low Complexity Regions and Universal Mask-GHI filters.

### Exome data analysis to detect somatic variants

VarScan2 variant detection algorithm version 2.4 [14] was applied to tumor-normal pairs from HNPCC20 to detect non-synonymous somatic variants from exome data. Exome sequencing data of HNPCC20 was received from FIMM in GRCh37 assembly. A .bam file from normal (blood sample) and matching tumor (FFPE sample) were used to create normal pileup and tumor pileup files by Samtools. Somatic analysis on output pileup files was with the following settings: tumor and normal purity 1.0, minimum somatic variant frequency (VAF) 0.05, minimum 2 reads in tumor, minimum coverage 8 in normal sample and 6 in tumor sample. Variants with a somatic p-value < 0.01 by Fisher’s exact test were considered significant. Somatic variant analyses of tumor-normal pairs from FCCX-U, FCCX-V, and FCCX-W were based on DRAGEN somatic mode with default parameters.

We applied the Java-based snpEff and SnpSift filters to annotate somatic variants from VarScan2-created snp.vcf and indel.vcf files (GRCh37.75 genome assembly) or DRAGEN-created sample.tn.hard-filtered.vcf (somatic variant) files (GRCh38.82 assembly). SnpEff annotations yielded a HGVS notation and sequence ontology terms for somatic variants [15]. After SnpEff, we used SnpSift Filter software to organize and filter somatic variants to select the most significant ones [16]. For HNPCC20, Ensembl Genome Browser 113 Tools Assembly Converter analysis followed to convert output files from GRCh37.75 to GRCh38.82.

### Somatic mutational signature analysis

We used the freely available web-based program Mutalisk (https://mutalisk.org) for mutational signature analysis. The analyses were based on VarScan2 output tumor.vcf file for family HNPCC20 and DRAGEN-based tn.hard.filtered.vcf tumor files for families FCCX-U, FCCX-V, and FCCX-W. Mutalisk generated an output figure for mutational signatures, number of total mutations, transcriptional strand bias, GC-content, and replication timing results per tumor sample [17].

### Loss of heterozygosity (LOH) analysis

The constitutional 3-bp deletion (HNPCC20) or insertion (FCCX-U and FCCX-V) in *BMPR1A* allowed variant-targeted LOH studies by fluorescent fragment analysis. Primers flanking the *BMPR1A* variant of HNPCC20 were 5’-[6FAM]GGCCATCTGTACCTGTTCACATTCA-3’ (forward) and 5’-TGGCCCCTCCCTTCTTTGTCT-3’ (reverse) and those for FCCX-U and FCCX-V 5’-[6FAM]TTCGATGGCTGGTTTTGCTC-3’ (forward) and 5’-ACAGCGGTTGACATCTAATA-3’ (reverse). LOH studies of FCCX-W were based on comparison of the abundance of constitutional *BMPR1A* missense variant-specific reads from the exome data of tumor vs. normal tissues by VarSeq and/or IGV (Integrative Genomics Viewer) analysis. The ratios of allelic peak areas in tumor DNA relative to normal DNA were interpreted to suggest strict LOH if equal to or below 0.60 or equal to or above 1.67, indicating that one of the alleles had decreased 40% or more, and putative LOH if between 0.6 and 0.8 or between 1.25 and 1.67, indicating a decrease of 21–39% for one allele [18].

### Haplotype analysis

To investigate a possible founding nature of the *BMPR1A* c.506-507insTCC insertion variant shared by families FCCX-U and FCCX-V, DNAs from a cancer-affected variant carrier of each family were genotyped with six microsatellite markers from the Ensembl database (pter-D10S219–D10S532–D10S1744–*BMPR1A*–D10S215 –D10S541–D10S185-qter). These markers spanned a 14.3-Mb region around the *BMPR1A* locus. Marker-specific PCR products were separated by gel electrophoresis and allelic fragments analyzed by GeneMapper software.

### Genealogical analysis

We used parish registers of the Finnish Evangelical-Lutheran church to trace the ancestors of FCCX-U and FCCX-V. Parish registers contain detailed information of births, marriages, and deaths, available from the beginning of the 17th century. By locating the birthplaces of the oldest known ancestors of the present-day families and searching for earlier ancestors one by one from the parish records and the web pages of the Finnish Genealogical Society [19], Geneanet [20], and My Heritage [21], we were able to trace a common ancestor couple for families U and V.

### In-silico analysis

To predict the effects of constitutional *BMPR1A* variants on BMPR1A protein, all the variant and wt amino acid sequences were inserted one by one into the SWISS-MODEL (https://www.expasy.org/resources/swiss-model) workspace [22] or AlphaFold Server (https://alphafoldserver.com/*)* [23] with default settings.

## Results

### Clinical features of *BMPR1A*-associated families

**Fig. 1:**
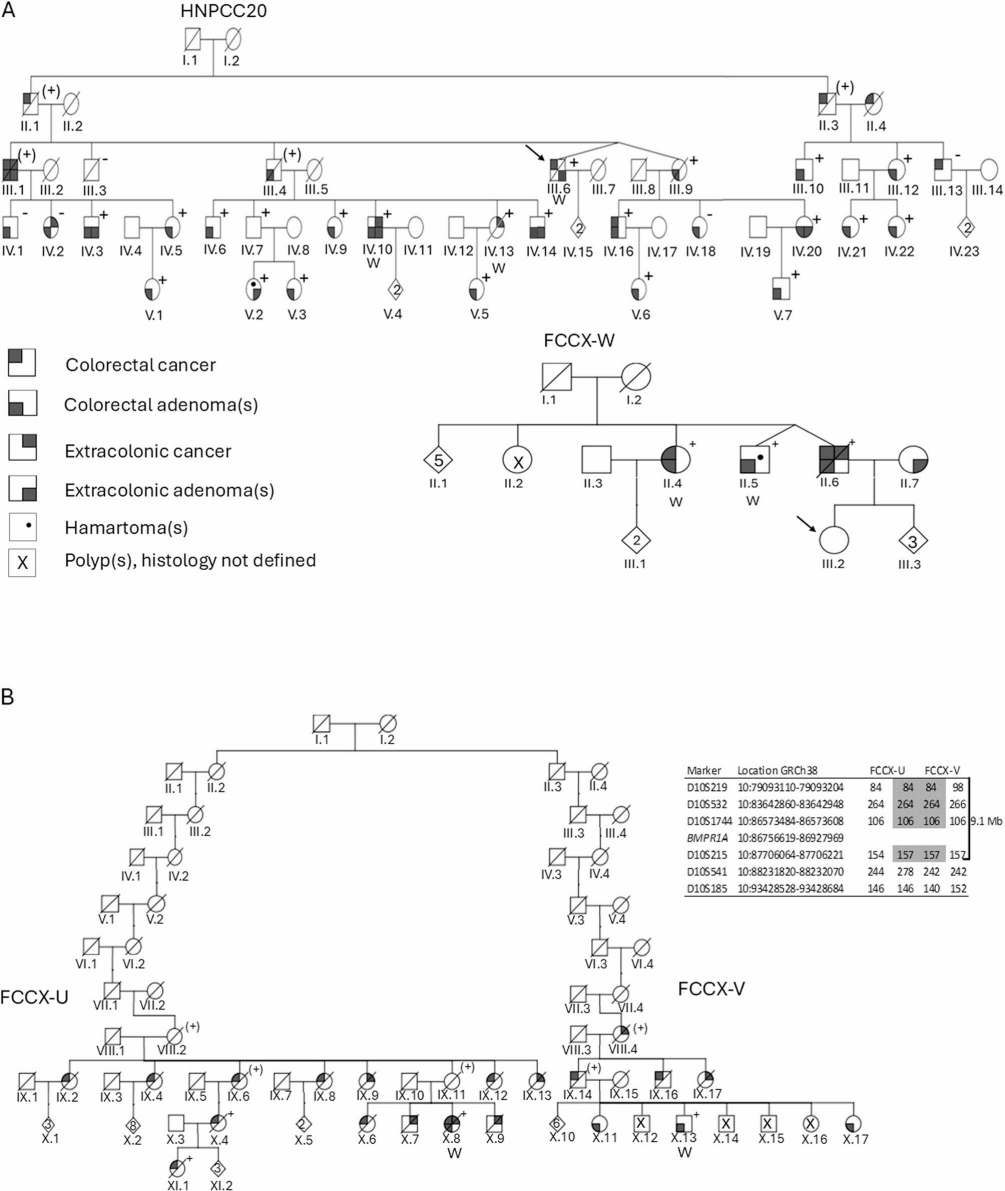
Pedigrees of families segregating non-truncating *BMPR1A* variants **A.** HNPCC20 (*BMPR1A* c.264_266del) and FCCX-W (*BMPR1A* c.766G > A). Numbers below the symbols are patient identifiers. Arrow denotes the index person (if known). A circle indicates a female and a square a male individual. Tumor manifestations are indicated by symbols explained on the left (arbitrarily drawn for a male individual). An open circle or square refers to the absence of any known neoplastic disease. A plus sign denotes the presence of the family-specific *BMPR1A* variant in a heterozygous state and a minus sign the absence of the variant. A plus sign in parentheses indicates obligatory carrier. Letter “W” identifies variant carriers whose samples (blood and/or tumor DNA) were included in whole-exome sequencing. Non-essential pedigree features have been excluded or modified to protect confidentiality. Pedigrees were drawn with CeGAT web site (https://cegat.com/) [42] **B**. FCCX-U and FCCX-V sharing the *BMPR1A* c.506_507insTCC variant. The families were found to descend from a common ancestor couple (generation I in the pedigree drawing) and share a haplotype of 9.1 Mb or more around *BMPR1A* (shaded portion in the haplotype image on the right). For pedigree symbols, please see Fig. 1A

We conducted a clinical and molecular investigation on four families clinically presenting as familial colorectal cancer with MMR-proficient tumors and diagnosed with heterozygous non-truncating *BMPR1A* variants by targeted sequencing (Fig. 1A and B). The *BMPR1A* c.264_266del p.(Glu88del) variant of HNPCC20 was originally published in Nieminen et al. [7], and we now provide an update. Moreover, we describe three families ascertained through clinical sequencing; these families, too, had non-truncating *BMPR1A* variants associated with adenomatous polyps. Families FCCX-U and FCCX-V shared an identical *BMPR1A* c.506_507insTCC p.(Ile169_Phe170insPro) variant, whereas a missense change (*BMPR1A* c.766G > A p.(Glu256Lys)) was present in family FCCX-W. Reference sequence for *BMPR1A* was NM_004329.3.

As illustrated by Fig. 1A and B, the *BMPR1A* variants co-segregated with colorectal cancer and/or polyps in the respective families. The clinical phenotypes of the patients are described in more detail in Supplementary Table 1. The age of first colorectal carcinoma diagnosis in *BMPR1A* variant carriers from these four families ranged from 48 to 71 years (average 62.1 years). In variant carriers, the highest number of polyps per colonoscopy was 5 and the largest number of cumulative polyps 25 (based on 11 colonoscopies). When this study began, no hamartomatous polyps were known to occur in any family. Upon histopathological re-review, all polyps examined had adenomatous histology, except for two variant carriers from HNPCC20 who were diagnosed with sessile serrated polyps of the colon (individual IV.3 had one and V.3 two such polyps), as well as individual V.2 from HNPCC20 and individual II.5 from family FCCX-W who revealed hamartomatous polyps. Among 25 proven or obligatory *BMPR1A* variant carriers with gastrointestinal polyps or cancer from HNPCC20, individual V.2 (who was not included in our original investigation [7]) showed a juvenile polyp in the gastric corpus at ~ 40 years of age. Among two *BMPR1A* variant carriers with gastrointestinal polyps or cancer from FCCX-W, two hamartomatous gastric polyps at ~ 80 years of age were found in individual II.5 on histopathological scrutiny, in addition to colonic adenomas. None of the six proven or obligatory *BMPR1A* variant carriers with gastrointestinal polyps or cancer from families FCCX-U/V had hamartomatous polyps. The small intestine was affected with carcinoma(s) or adenomas in five *BMPR1A* variant carriers, all from HNPCC20 (III.1, III.6, IV.10, IV.13, and IV.20). Gastric polyps were uncommon: apart from the two individuals with hamartomatous polyps described above, individuals IV.3 and IV.14 from HNPCC20 were diagnosed with fundus gland polyps.

### Characteristics of constitutional *BMPR1A* variants

**Fig. 2:**
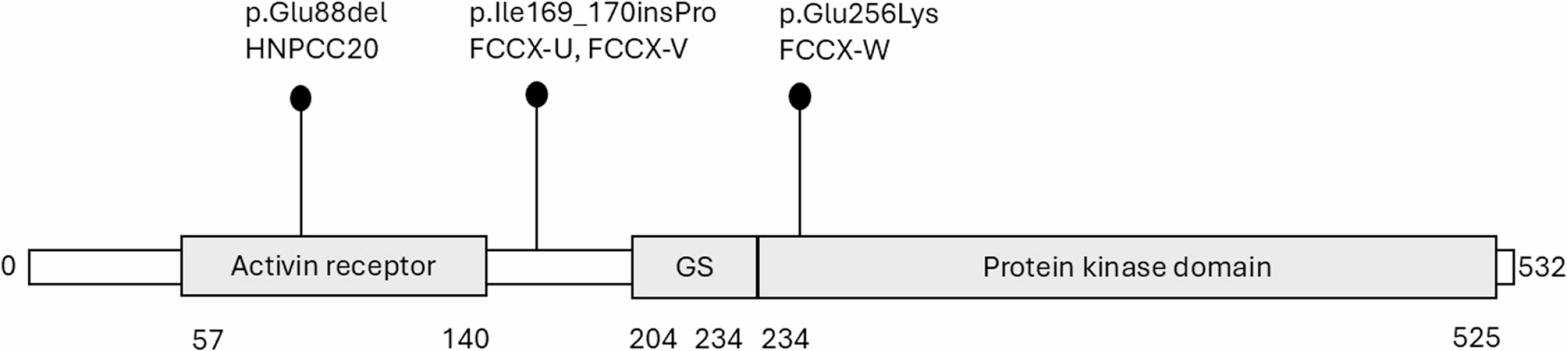
Lollipop diagram indicating the location of the constitutional *BMPR1A* variants against the main functional domains of the encoded protein, namely activin receptor, GS domain [24], and protein kinase domain. Domain information was taken from the InterPro database (https://www.ebi.ac.uk/interpro/) [43]

The constitutional alterations detected were distributed throughout the BMPR1A protein (Fig. 2). The extracellular, ligand-binding domain was affected in HNPCC20 and intracellular kinase domain in FCCX-W, whereas the variant shared by FCCX-U and FCCX-V was located between established functional domains [24]. SWISS-MODEL (Suppl. Figure 1 A) and/or AlphaFold modeling (Suppl. Figure 1B) predicted abnormal three-dimensional configuration of each mutant BMPR1A protein. All three variants have previously been reported two to four times to the ClinVar [25] database with hereditary cancer-predisposing syndrome or JPS as clinical diagnoses and classified as variants of uncertain significance (VUS). The variants are very rare or absent in the general population (allele frequencies below 0.0001 in Finns and all populations, no homozygotes, according to GnomAD v.4.1 [26]).

*BMPR1A* variant sharing between FCCX-U and FCCX-V, combined with the rarity of the variant in the average population, prompted us to explore the ancestral origin of the families by haplotype and genealogical analyses (Fig. 1B). Haplotype analysis on the index individuals revealed a shared haplotype of at least 9.1 Mb around *BMPR1A*. Genealogical analysis identified a common ancestor couple eight generations back from generation IX of the present-day families. This ancestor couple had lived in the Northern Ostrobothnia region of Finland in the late 17th century. These findings allowed us to conclude that families FCCX-U and FCCX-V originated from a common ancestor.

To exclude other possible predisposing variants beyond *BMPR1A*, we conducted exome sequencing on 2–3 members with colorectal cancer and/or polyps from each family. Variants that met our inclusion criteria (see Materials and Methods) and were shared between the affected members of each family are listed in Supplementary Table 2. Apart from the *BMPR1A* variants described above, no pathogenic or likely pathogenic variants in known cancer susceptibility genes were identified in any individual by exome sequencing. Moreover, no such variants in possible novel candidate genes were present that could be considered as plausible predisposing variants for the families.

### Two-hit inactivation of *BMPR1A* in tumors

**Fig. 3:**
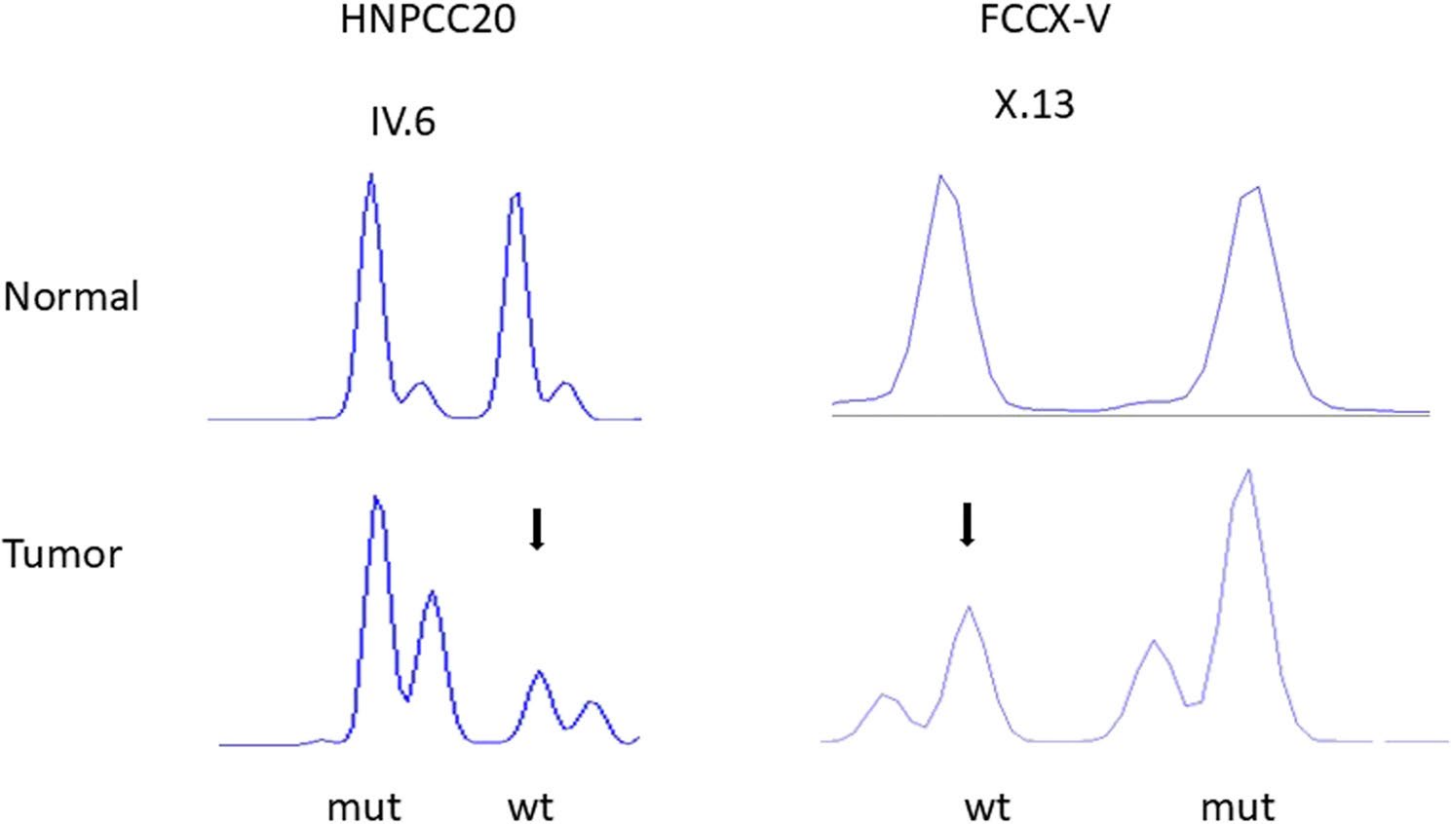
LOH analysis of colorectal adenomas from families HNPCC20 and FCCX-V. The constitutional *BMPR1A* variants (3-bp deletion in HNPCC20 and 3-bp insertion in FCCX-V) were used as intragenic markers in fragment analysis. Colonic adenomas from individual IV.6/HNPCC20 (left) and X.13/FCCX-V (right) showed wild-type allele LOH with LOH ratio of 0.33 and 0.48, respectively. “Wt” refers to wild-type and “mut” mutant allele. Attenuated alleles are marked with arrows

All available tumors from *BMPR1A* variant carriers were retrieved from pathology archives and DNAs extracted for studies of somatic alterations. To address LOH at the *BMPR1A* locus, we took advantage of the three-nucleotide deletion (HNPCC20) or insertion (FCCX-U and FCCX-V) to separate PCR products specific to the wild-type (wt) and mutant alleles by fragment analysis (Fig. 3). DNAs of 20 intestinal tumors (mostly adenomas) were available from *BMPR1A* variant carriers of HNPCC20, and among these, 3 tubular adenomas (one from IV.22 and two from IV.6) showed wt LOH (15%). The single colorectal adenoma available from FCCX-U (individual X.8) revealed no LOH, whereas one of three adenomas from FCCX-V (individual X.13) had wt LOH. In FCCX-W, the predisposing *BMPR1A* variant was a missense change, and we utilized exome sequencing data for LOH analysis by comparing the reference to mutant allele ratios in tumor and normal DNA. Two adenomas (individuals II.5 and II.4) showed wt LOH, whereas colorectal carcinoma (individual II.4) had retained heterozygosity. Owing to insufficient amounts of DNA, we were unable to perform LOH analysis on the two gastric polyps with hamartomatous features from individual II.5 of FCCX-W.

Somatic point mutations of *BMPR1A* as possible second “hits” were screened for by exome sequencing. One such event was found: a colorectal adenoma from FCCX-U (individual X.8) revealed a six-nucleotide deletion (c.334-2_337delAGGATT) affecting the splice acceptor of *BMPR1A* exon 4 and having a variant allele frequency of 21% (Supplementary Table 3). Taken together, two-hit inactivation by either LOH or somatic point mutation was observed in 3/20 tumors (15%) from HNPCC20, 2/4 tumors (50%) from FCCX-U/V and 2/3 tumors (67%) from FCCX-W.

### Overall somatic mutational profiles and signatures

**Fig. 4:**
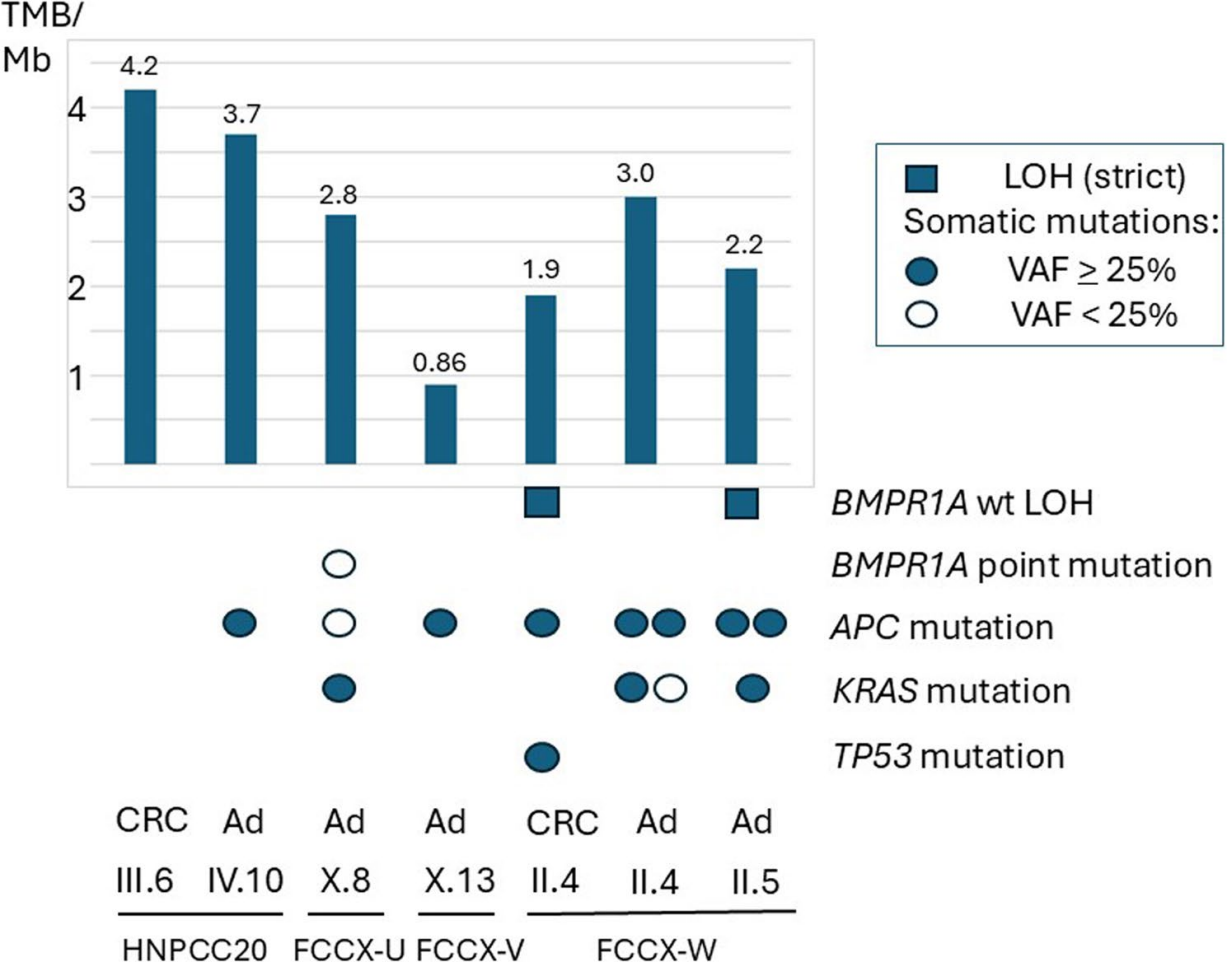
Schematic summary of key somatic mutational features of tumors from *BMPR1A* variant carriers. Results from the seven tumors included in exome sequencing are shown. CRC denotes colorectal carcinoma and Ad adenoma. Of the five adenomas investigated, four were colorectal (from individuals X.8 of FCCX-U, X.13 of FCCX-V, and II.4 and II.5 of FCCX-W) and one jejunal (from individual IV.10 of HNPCC20). Bar graph indicates tumor mutational burden (TMB), expressed as the total number of nonsynonymous somatic mutations per megabase. Two-hit inactivation of *BMPR1A* is depicted by squares (LOH) and circles (somatic point mutations). *APC*, *KRAS*, and *TP53* mutations (circles) represent typical colorectal cancer drivers (please see Supplementary Table 3 for details of the mutations)

All somatic variants fulfilling our selection criteria and detected by DRAGEN somatic analysis (FCCX-U, FCCX-V, and FCCX-W) or VarScan2 analysis (HNPCC20) from exome sequencing of tumor tissues are listed in Supplementary Table 3. Consistent with mismatch repair proficiency by MSI and/or immunohistochemical analyses performed on the families as part of their initial diagnostic workout, none of the seven tumors we studied, two carcinomas and five adenomas, was hypermutated by exome sequencing. The tumor mutational burdens (TMB) varied between 0.86 and 4.2/Mb, being clearly below the hypermutability threshold of 10 nonsynonymous somatic mutations per Mb (Fig. 4). The top mutant genes (proportion of tumors mutant out of seven in parentheses) were *APC* (6/7, 86%), *EPPK1* and *FSIP2* (4/7, 57%), and *APCDD1L*, C22orf42, *KRAS*, *KRT34*, *NBPF9*, *RNF213*, and *TTN* (3/7, 43%). Typical of MMR-proficient colorectal adenocarcinomas (and adenomas) in general [5], *APC* was mutant in all tumors but one (colorectal carcinoma of individual III.6 from HNPCC20), and *KRAS*, too, was frequently affected (Fig. 4; Supplementary Table 3). *TP53* was mutant in one tumor (colorectal carcinoma of individual II.4 from FCCX-W).

Analysis of COSMIC [27] single-base substitution (SBS) signatures v.3.4 revealed no distinctive features in tumors from *BMPR1A* variant carriers. SBS1 (spontaneous deamination of 5-methylcytosine, clock-like signature) predominated, occurring in all seven tumors analyzed (Supplementary Table 4).

## Discussion

The clinical diagnosis of JPS requires five or more juvenile polyps in the colorectum, juvenile polyps throughout the gastrointestinal tract or any number of juvenile polyps and a positive family history of juvenile polyposis [6]. Atypical histological features of polyps and complex and variable clinical manifestations may lead to misdiagnoses [3, 28]. The clinical diagnosis of JPS can be confirmed in approximately half of the patients by the identification of pathogenic variants in *BMPR1A* or *SMAD4* [3]. On the other hand, constitutional variants of especially *BMPR1A* can result in clinical and histological phenotypes other than JPS. We, here, scrutinized four families segregating non-truncating *BMPR1A* variants and having clinical presentation of familial non-polypotic colorectal cancer or FCCTX. Our findings from genetic analyses of constitutional and tumor tissues and histological evaluation of tumor tissues highlight the complex genotype-phenotype relationships of *BMPR1A* variants.

Phenotypes other than JPS reported in association with *BMPR1A* variants to date include FCCTX [7, 29] this study, early-onset colorectal cancer with mismatch-repair proficiency [30], attenuated familial adenomatous polyposis [28, 31]– [32], hereditary mixed polyposis syndrome type 2 [33–35], and combined features of more than one of the above [36]. Polyps in these cases have been adenomatous, or in the case of hereditary mixed polyposis syndrome, included juvenile, adenomatous, and hyperplastic polyps.

Analysis of patients fulfilling the clinical criteria for JPS and having pathogenic variants in *BMPR1A* has shown that in addition to juvenile polyps, up to half of the carriers may have other types of polyps, too, such as adenomatous or serrated polyps [3]. Multiple microsatellite-stable colorectal adenomas and carcinomas were the predominant characteristic of the four families we studied. HNPCC20 fulfilled the Amsterdam criteria for hereditary non-polyposis colorectal cancer [8]– [9], and one individual (V.2 in Fig. 1A) was diagnosed with a juvenile polyp in gastric corpus. FCCX-U and FCCX-V were united to a single family through a common ancestor and also fulfilled the Amsterdam criteria if the requirement of diagnosis at early age was not considered; these families showed no hamartomatous polyps. Contrary to the large families HNPCC20 and FCCX-U/V, FCCX-W was small and met the Bethesda criteria [10]. One individual from FCCX-W (II.5 in Fig. 1A) had gastric polyps with hamartomatous features.

Possible explanations for different phenotypes associated with constitutionally mutant *BMPR1A* alleles include at least location and type of constitutional variant, somatic changes in tumor tissues, possible modifying constitutional variants, and environmental influences. In JPS patients, pathogenic *BMPR1A* variants are distributed across all functional domains of the gene and can be of frameshift (39%), nonsense (24%), or missense type (18%), or large deletions (3%) [37]. Variant location or type does not appear to correlate with disease severity [37]. The *BMPR1A* variants of our present four families affected coding exon 3 (extracellular, ligand-binding domain) in HNPCC20, coding exon 5 (no functional domain) in FCCX-U and FCCX-V, and coding exon 7 (intracellular, kinase domain) in FCCX-W. All variants were non-truncating: that of HNPCC20 was a 3-bp deletion, that of FCCX-U and FCCX-V a 3-bp insertion, and that of FCCX-W a missense change. Each variant has previously been reported to ClinVar [24] two to four times, with diagnoses of hereditary cancer-predisposing syndrome or JPS and classified as VUSes. In our families, the following ACMG/AMP criteria [38] were met: co-segregation of the variants with colorectal cancer and/or polyps (Fig. 1A and B) (PP1), aberrant predictions by in-silico modeling (Suppl. Figure 1) (PP3), and rarity (allele frequencies below 0.0001) in the average population (PM2). Additionally, two-hit inactivation in tumors (Fig. 3) provided evidence of loss of function. While all these findings supported the idea that the variants were deleterious and underlay tumor predisposition in the associated families, the variants formally remained in the VUS category according to the ACMG/AMP classification.

The requirement and mechanisms of “two-hit inactivation” in *BMPR1A*-associated tumorigenesis remain controversial. Jacoby RF et al. [39] detected somatic deletions at 10q22, the site of *BMPR1A*, in 39/47 juvenile polyps (83%) representing either hereditary or sporadic forms. By FISH, the deletions affected exclusively stromal cells (lamina propria) and not epithelial cells. Blatter et al. [40] investigated 11 polyps from carriers of a constitutional nonsense variant of *BMPR1A* (c.583 C > T, p.(Gln195*)) and found that the wild-type allele of *BMPR1A* was lost in 5/9 juvenile polyps (56%) but present in 2/2 adenomas. Studies of laser-capture-microdissected cells demonstrated that LOH occurred in the epithelial compartment and was absent in stroma, opposite to the findings by Jacoby et al. [39]. Moreover, LOH was copy-number neutral suggesting homologous recombination as a possible mechanism [40]. Our LOH studies were based on bulk DNA, precluding any cell-type specific assessment. Apart from LOH, we addressed somatic *BMPR1A* point mutations, too, as possible inactivating “second hits” in adenomas and carcinomas from our *BMPR1A*-associated families. We were able to demonstrate two-hit inactivation in intestinal (colon) tumors, mostly adenomas, in 15% (3/20) of tumors from HNPCC20, 50% (2/4) of tumors from FCCX-U/V, and 67% (2/3) of tumors from FCCX-W. These somatic events consisted of wt LOH (all families) and a splicing variant (FCCX-U). It is possible that two-hit inactivation is not necessary for tumorigenesis in all cases, since a Drosophila model of JPS-associated *BMPR1A* mutations revealed that mutations with no phenotype in heterozygous carriers exhibited tissue-level effects when present as sporadic clones of somatic cells at a heterozygous state [41]. This phenomenon, termed “deleterious heteromosaicism”, suggested a one-hit mechanism for disease initiation.

BMP signaling involves multiple ligands, multiple ligand-sequestering antagonists, multiple receptors, and multiple downstream effectors [3], and different combinations of interacting molecules and their altered balance resulting from constitutional or acquired changes may explain the complex genotype-phenotype relationships of the associated disorders to a significant extent. Apart from the *BMPR1A* variants, exome sequencing of constitutional DNAs did not reveal other likely candidates for predisposing variants among those shared by at least two affected members of our families (Supplementary Table 2). A possible phenotype-modifying role for these additional variants cannot be ruled out, although the respective genes involved only one family each. Finally, no single prominent or consistent signature stood out by somatic mutational signature analysis (Supplementary Table 4); however, within each family, the signatures of different tumor samples resembled each other, which may point to shared environmental or endogenous etiologies.

## Conclusions

In summary, we describe four families with non-truncating *BMPR1A* variants exhibiting FCCTX or FCCTX-like phenotype, whereas the ClinVar database includes individual reports of the same variants associated with JPS. Since the number of available reports of *BMPR1A* variants presenting with FCCTX or FCCTX-like phenotype is limited (see above), the phenotypic association we propose requires confirmation from additional studies. The non-truncating nature of the variants does not appear critical in this context since non-JPS phenotypes characterized by adenomatous polyps may likewise result from truncating *BMPR1A* variants [7, 28, 30, 31]– [32]. Our investigation, together with previous reports, highlights the multiplicity of histological, clinical, and family features that may be associated with constitutional *BMPR1A* variants, awareness of which is important in the clinical management of variant carriers and their family members. While the exact basis of different phenotypes remains unknown, our molecular findings from constitutional and tumor tissues provide worthwhile hypotheses for further studies. Additionally, a mechanistic dissection of pathogenicity of the present non-truncating *BMPR1A* variants awaits future functional investigations.

## Supplementary Information

Supplementary Figure 1. Predicted three-dimensional structures of wild-type and mutant BMPR1A proteins. A. Configurations by SWISS-MODEL. Confidence level of prediction is indicated by color scale as shown. B. Configurations by AlphaFold. Confidence level of prediction is indicated by color scale as shown.

Supplementary Material 2.

Supplementary Material 3.

Supplementary Material 4.

Supplementary Material 5.

Supplementary Material 6.

## Data Availability

The datasets are available from the corresponding author on reasonable request.

## Abbreviations

ACMG/AMP: American College of Medical Genetics and Genomics/Association for Molecular Pathology
BMP: Bone morphogenetic protein
BMPR1A: Bone morphogenetic protein receptor type 1A
FCCTX: Familial colorectal cancer, type X
FFPE: Formalin-fixed paraffin-embedded
HGVS: Human Genome Variation Society
IGV: Integrative Genomics Viewer
JPS: Juvenile polyposis syndrome
LOH: Loss of heterozygosity
MMR: DNA mismatch repair
SBS: Single-base substitution
TMB: Tumor mutational burden
WT: Wild-type
