# Supplementary figures and images for: Non-truncating BMPR1A variants associated with familial colorectal cancer and adenomatous polyps
Source: BMC Cancer. 2025 Sep 29;25:1435. doi: 10.1186/s12885-025-14865-8 (PMC12481737; doi:10.1186/s12885-025-14865-8)

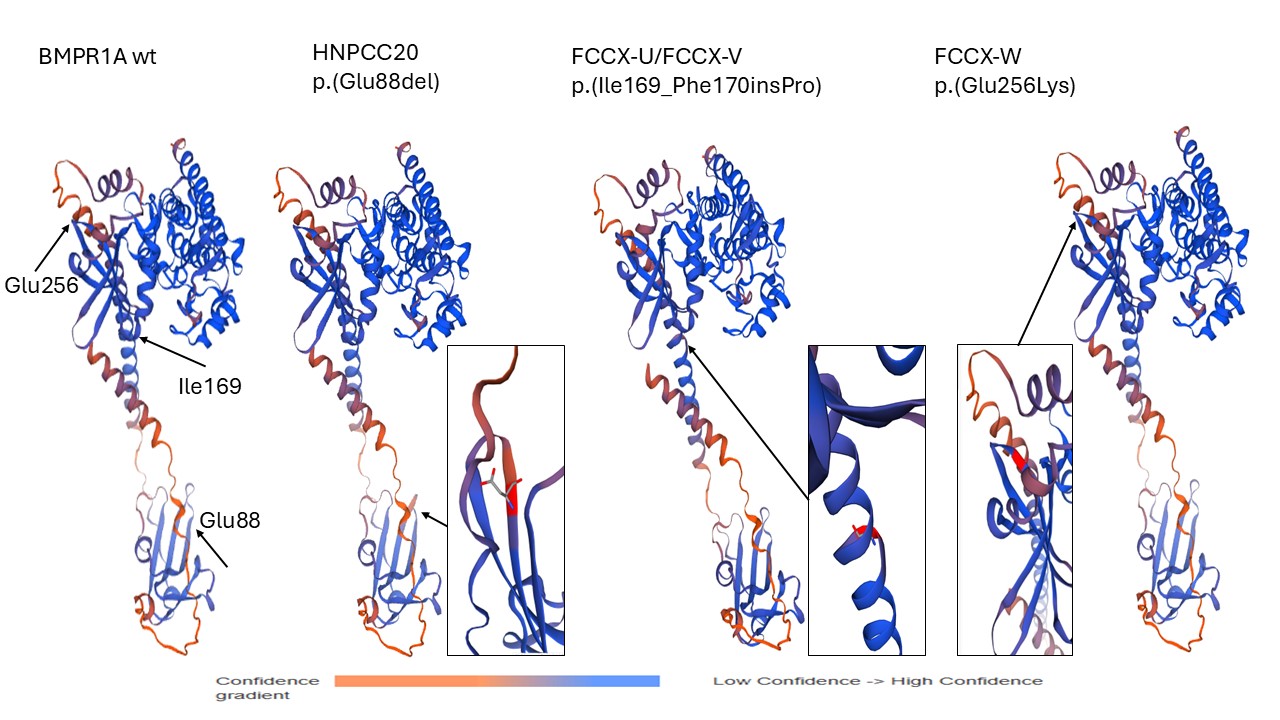

Supplement: Supplementary file 4 — Supplementary Material 4. [file 12885_2025_14865_MOESM4_ESM.jpg]

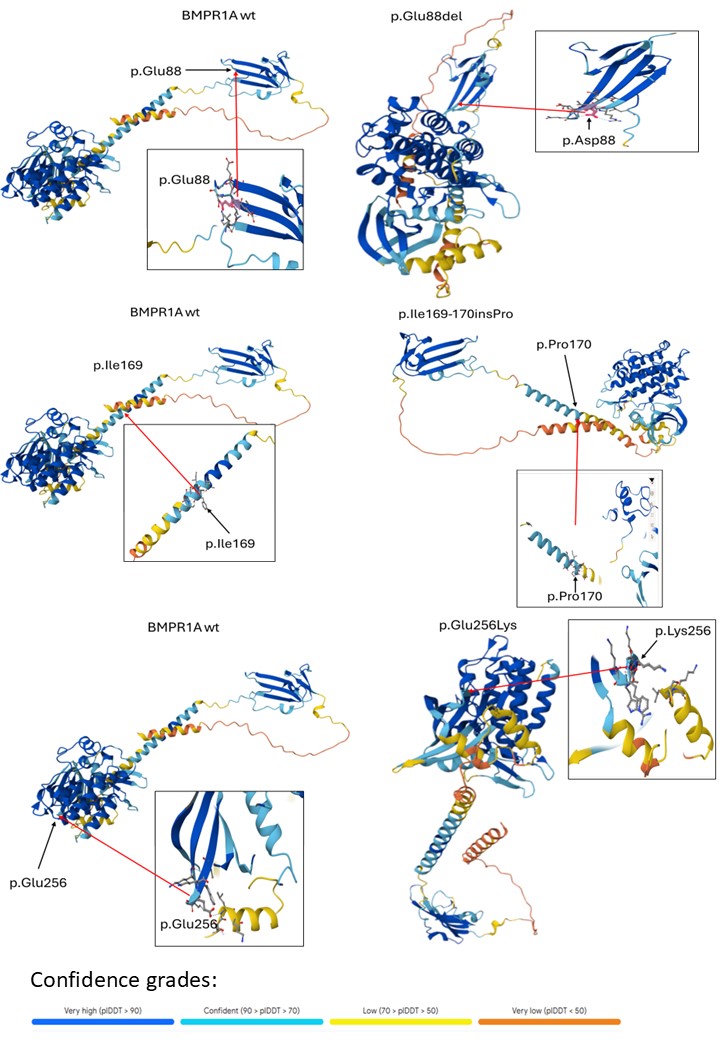

Supplement: Supplementary file 5 — Supplementary Material 5. [file 12885_2025_14865_MOESM5_ESM.jpg]
